# Towards an open science publishing platform

**DOI:** 10.12688/f1000research.7968.1

**Published:** 2016-02-03

**Authors:** Vitek Tracz, Rebecca Lawrence

**Affiliations:** 1 F1000, London, UK

**Keywords:** open science platform, preprint, impact factor, open access, open data, life sciences, publishing

## Abstract

The way science and research is done is rapidly becoming more open and collaborative. The traditional way of publishing new findings in journals is becoming increasingly outdated and no longer serves the needs of much of science. Whilst preprints can bring significant benefits of removing delay and selection, they do not go far enough if simply implemented alongside the existing journal system. We propose that we need a new approach, an Open Science Platform, that takes the benefits of preprints but adds formal, invited, and transparent post-publication peer review. This bypasses the problems of the current journal system and, in doing so, moves the evaluation of research and researchers away from the journal-based Impact Factor and towards a fairer system of article-based qualitative and quantitative indicators. In the long term, it should be irrelevant where a researcher publishes their findings. What is important is that research is shared and made available without delay within a framework that encourages quality standards and requires all players in the research community to work as collaborators.

## Introduction

The way science and research is done is evolving rapidly. The change is characterised by more open, collaborative and networked ways of sharing information and making discoveries. This change is being driven by recognition of the profound benefits to the pace of scientific progress that can be brought by collaboration and ready exchange of ideas between and beyond disciplines and sectors. Furthermore, technology can now enable and support collaboration, information sharing and rapid data exchange and analysis. To make science more efficient, we need to remove the waste in the current system, as exemplified by ongoing debates concerning research duplication and the growing doubts about the reproducibility of findings.

The goal for open science is to accelerate scientific progress and to turn what is discovered into benefits for all. An essential part of this is to ensure that scientific findings are open and available for scrutiny, rapidly accessible, and easily discoverable for others to use and build upon. The way research findings are currently made available – through journals – is increasingly at odds with the aspirations of open science.

## Journals: an outdated mechanism for publishing work

There is some recognition amongst the research community that journals are now an outdated method for publishing new research findings and no longer serve the needs of much science ^1, 2^. The current publishing processes bring many problems that are not conducive to the progress of science. These include:

• **Holding up science.** The selection process that journals run to help them decide what to accept is typically done before publication, and hence leads to a delay in the availability of new findings to those who need them ^3^. There are no obvious benefits of such a delay, and indeed it can sometimes cause significant damage when the health of patients are affected ^4^.
• **Non-transparent.** The peer review scheme used by most journals is anonymous (the choice of referees is hidden from the authors, and the referees’ comments are not always fully shared with the authors). Its current main function is usually to help editors make decisions on what to publish and what to reject, rather than to help the authors improve their article. Furthermore, the readers do not get the benefit of the insight on any outstanding issues the referees may have identified with the article along the way. In a competitive research environment, these non-transparent schemes can lead to abuse of the system in a variety of different ways ^5, 6^ and there is little to stop this from happening.
• **Much science never shared.** Many findings are currently not published (such as small studies, data and software papers, negative and null studies etc), which often leads to significant research waste and potential publication bias ^7– 9^. This is often caused by the fact that journals, in order to maintain their Impact Factor, are keen to attract submissions that bring in more citations ^10^.
• **Waste in the system.** There is significant waste in the publishing system caused by articles moving from journal to journal until they find somewhere that will accept to publish the article. This brings inefficiencies in the system and wasted effort both for the authors and for the referees in repeated refereeing.
• **Too expensive.** Most new scientific findings are still published in subscription journals that are usually expensive. This means that a large proportion of the community (researchers and the public) cannot access the research ^11, 12^. Open access journals certainly enable everyone to have access but their Article Processing Charges are still often very high ^13^. So-called hybrid journals (subscription journals that require authors to pay for an open access option) are even worse, because they create extra costs for the information exchange system and significantly slow the growth of fully open access journals.

Which journal an article is published in is still commonly used as a surrogate quality measure for an individual article and its authors. However, it is well established that such use, specifically of a journal’s Impact Factor (JIF), is an inappropriate and misleading indicator of either the importance and/or quality of a specific article ^14– 17^, or of the potential of the author(s) as researchers ^18^.

The reality is that journals are not essential anymore (though sometimes useful) for the discovery of research results. Much more efficient tools and services can and are being developed using the information in citation databases such as PubMed (for biomedical research) and/or Google Scholar, Scopus, Web of Science etc., to help researchers find new articles in an area of interest. Journals survive primarily because they are needed by authors to get the reflected benefit of the JIF. One of the challenges for the future is to develop reliable and effective qualitative assessment of both research articles and an individual researcher’s scientific output.

A new way of publishing, discussing and reviewing new scientific findings is urgently needed to speed up the progression of science, and to improve the fairness of the system used to judge researchers with regards to their next grant or career move. Such a new system should also enable funders to maximise the value of their research investment. The technology to enable such a change is now available. This new approach needs to avoid the significant delays in making new findings visible, and needs to be efficient, easy to use and not expensive.

In our view, and as we outline further below, such a new process would only work if driven by the authors within a scientific framework that facilitates self-regulation. There needs to be a generally agreed set of ethical and technical rules, and these should be overseen by bodies directly representing researchers, such as funders, institutions, organisations and societies.

## Preprints: the benefits and limitations

One idea currently being discussed is the much wider use of preprints in the life sciences, i.e. the online posting of an article to make it openly visible and citable prior to peer review ^19– 21^. One of the features of preprints is that authors remain completely in control and no formal refereeing is required. The culture of using preprint servers in physics ( arXiv) has established itself well without structured refereeing. It is surprising that preprints and journals have survived alongside each other for so long, and that authors still feel the need to have the preprint converted into an article published in a journal, even though all who need to have already accessed the article from the preprint server. It seems that even here, where the article is published can have a significant impact on the prospects of the authors’ career. This perhaps illustrates the powerful hold that journals and JIFs have on researchers’ careers. Preprints, even when used widely, may not remove this dependence.

Despite a modest increase in the use of preprints in life sciences recently with the launch of PeerJ PrePrints and BioRxiv, the overall use of preprints to-date in the life sciences is still not in significantly large numbers (under 5,000 preprints posted on these two sites to-date, compared with over 1.13 million articles added to PubMed just in the past year). There have been several attempts to encourage the use of preprint servers, but none has really taken off. In the early days of open access back in the late 1990s, the idea had been to start with a preprint server, but there was such strong objection from some members of the PubMed Central (PMC) National Advisory Committee in its first meeting ^22^, that a decision was taken not to accept preprints. The prevailing culture within the research community at the time was not to recognise preprints as a formal output, and it is not clear how different the culture really is now.

There are many benefits to authors and to the community of using preprint servers as part of the mix of options in publishing life science research articles. Perhaps most important is the immediacy of placing research findings on public record (also thereby establishing some level of priority). Preprints can also be used to gather informal comments from colleagues on possible problems and potential improvements before committing to the non-transparent refereeing as operated by most journals. But preprints solve only some of the problems outlined earlier, and technology now enables us to do much more with findings at the stage that researchers are willing to share them.

Many questions have yet to be answered about the adoption of preprints in the life sciences. Among them:

• Will a significant proportion of authors opt to post preprints?
• Will enough colleagues and other researchers comment openly (or even confidentially) on articles posted there, to make the effort useful? A quick review of a randomly selected block of 100 articles posted on BioRxiv in June/July 2015 (so over 6 months ago to ensure time to receive comments) showed that there were only two preprints in that selection that had any external comments, each being back and forth with a single commenter.
• Will subsequent formal submission to a journal and progress through the lengthy process of official peer review as currently operated by journals still be required, thereby retaining many of the problems outlined earlier?

What we propose here is a scheme that takes full advantage of the benefits that preprints can bring, combined with a new type of invited, formal transparent peer review that differs significantly from the one currently run by most journals, both in its goals and in its processes. The description of this scheme (which has been running for three years now in the publication process of F1000Research) is described below.

## A new experiment: Open Science Platform

Here we present a model as a starting point that we envisage will evolve as researchers embrace the opportunity to share their findings and data in new ways. Our guiding principle is that open science publishing should be author-driven to enable researchers to share openly and rapidly any new findings that they think are worth sharing. Findings should be published near immediately, in a format most appropriate to convey the information in the discovery. In addition, publication should be usually followed by post publication, formal invited peer review, that is conducted transparently. This is both to help authors to improve their presentation and to provide auditable qualitative assessment of the research.

**Writing and submission.** The process of compiling findings, writing accompanying narrative and making this available for public view and scrutiny can be simplified by the use of new improved software. These tools can help identify relevant papers through increasingly powerful learning algorithms (e.g. F1000Workspace, Mendeley, Readcube). They can also enable collaborative authoring (e.g. F1000Workspace, Overleaf, Google docs), and provide formatting tools to simplify the process of structuring an article to ensure all the necessary underlying information has been captured (e.g. F1000Workspace, EndNote). Submission for posting as a preprint, and/or for formal publication and peer review, should be as simple as a single click.

**Initial objective checks.** We envisage that all submitted articles should be rapidly screened against a set of objective criteria. Such criteria might include checks for obvious non-scientific content, readability, ensuring the work is not plagiarised, that it meets standard ethical requirements, and that the underlying data has been supplied together with detailed methods. They could also include other specifics as agreed by the relevant communities depending on, for example, the type of experimental study being described. The specifics of these checks should be listed transparently.

**FAIR (Findable, Accessible, Interoperable and Reusable) data.** The need for an open data policy seems undisputable to us: the data underlying the findings presented in the article should be openly accessible, together with information on how the data were analysed such as the software used etc., so that users can fully scrutinise the presented findings and repeat the work, if they wish. The data and any code should be in a form that can be used by the referees and readers, together with detailed methods as to how it was generated. They need to be stored in approved repositories that meet a minimum set of criteria to ensure long-term availability and persistence, with appropriate levels of protection for sensitive data. How much and what data to provide is a highly complex issue and will require specific instructions to be developed by the relevant communities, together with more generalised requirements on data format, structure and associated metadata. There are numerous groups working collaboratively worldwide on these many issues such as the Data Fairport Initiative, FORCE11, Research Data Alliance and others.

**Publication.** Any submitted article that passes these rapid checks would then be published (made public) immediately, given a unique identifier (making them permanently citable) and clearly labelled as not yet peer reviewed. Following the initial screening, we think it is important that there is no editorial decision on accepting or rejecting research articles, to remove the inherent biases in having a single Editor making a decision on behalf of the rest of the community, and to help to remove publication bias.

**Identifying referees.** In order to facilitate communication between peers without the interference of editors, and to ensure peer review is carried out by qualified experts, we envisage that authors should select referees from a large community of recognised experts (potentially with the assistance of algorithmic tools), as long as they abide by a clear set of transparent rules and criteria on how to select suitable referees. Both authors and referees should also transparently declare any conflicts they have with each other or the work being refereed.

Questions remain around how this community should be defined. Should a database (growing and changing) be created of ‘approved referees’? How does someone qualify to be included in this community of approved referees? How will this database grow, and who will control it? For example, it could comprise grantees of major granting bodies with some minimum experience/publication record. Or it could be constructed like a large virtual faculty like the F1000 Faculty. It would seem that there should be greater collaboration across the key stakeholders involved in research (funders, publishers, research institutions, researchers, industry) to work together to resolve these issues.

**The peer review process.** Referees should then be invited as requests from the authors, but mediated by the platform. Without the need to select for impact, the peer review process can refocus on its basic goal to help the authors improve their work and to provide valuable context and feedback on the viability and quality of the published research for the reader and for anyone reviewing the work of that individual.

Referees should be given a set of clear instructions and guidance on what aspects of the article to assess and what is expected in a referee report (as common practice today). Referees should also be able to benefit from tools similar to those provided to authors, to make the writing of the report more efficient. With the publication of new types of findings, there is an interesting question about whether all findings need to be refereed (for example, short commentary articles). There are also many questions about what types of aspects of an article can a referee sensibly be expected to check within a reasonable timeframe, especially with regards to data, code and figures. Should peer review differ for different types of findings?

Given the open questions about what level of peer review should be required for different article types and for data and software, there are also questions around what constitutes an article being ‘peer reviewed’. PubMed has developed criteria for F1000Research and future publishing platforms ^23^, but should this always be the same irrespective of the type of finding?

We think it is very important that all peer review reports are published transparently alongside the name of the referee – open peer review has been repeatedly shown to be of comparable (if not better) quality, and also often more constructive compared with closed peer review ^24, 25^. The authors would drive the process via the platform provider, so that they can engage in open discussion with the referees and can revise their article and publish new findings as and when they feel appropriate. This process should continue until the authors wish to stop. All versions need to be independently citable but connected, and a dynamic citation ^26^ can be used to ensure the reader is always clear about the article version and its peer review status. In addition to formal refereeing, any researcher should be able to openly comment and discuss an article in a transparent way, although this should not impact the formal peer review status of the article.

**Benefits for referees.** Referees currently receive very little direct benefit from the process of refereeing and their contribution is currently not visible. We believe referees should receive real benefits for contributing to what is a crucial function in improving the work of others. Referee reports should receive their own persistent identifier (digital object identifier – DOI) and therefore be independently citable which means that referees can receive their own metrics. Refereeing can now be included as a formal contribution on Publons and on ORCID profiles ^27^ (the researcher unique persistent digital identifier), and we would urge institutions and funders to lay out an expectation (and provide formal recognition), for their grantees to contribute to this important process. Are there other forms of credit that referees could receive for their important role? Should ways be developed to qualitatively assess the work of referees?

**Access and cost.** All articles should be published (made visible and citable) using immediate open access so that everyone has equal access to new findings. The cost of running the process described above is considerably cheaper than the traditional process as it removes the substantial costs associated with editorial decision making. There are of course still costs involved in running the peer review process, conducting the initial set of checks, and building and maintaining the tools required to operate such a system. These cheaper costs would still be covered as now by research funders, and competition between service providers should put further downward pressure on these costs. Where there is no research funding, there needs to be further consideration across all stakeholders as to how best to cover the modest fees, whether through institutional funds or other sources.

## Indicators of quality and importance

It remains important that there are indicators of the value, importance, use and re-use of research findings and data. Research outputs, in all their forms, are valuable indicators of research and knowledge progression, as well as of the ‘performance’ and productivity of scientific fields and of the researchers who are generating those outputs. Such indicators are also vital for users of research findings, such as health professionals and policy makers, to help get relevant research findings into policy and practice more effectively and without unnecessary delay.

The indicators that are adopted to provide a view on research must be meaningful, contextualised and used responsibly ^28^. The Leiden Manifesto ^29^ recently emphasised the importance of combining quantitative and qualitative indicators in assuring a balanced and robust conclusion about the value of specific research. Furthermore, the selection of indicators that are used in any assessment should be tailored according to the purpose of the assessment. We should all seize the opportunity of working in a different publishing system to respond to recommendations such as those in the Manifesto, and redress our reliance on erroneous and misleading measures of research quality. Improvements in our ability to identify, track and analyse the outputs means that we can also shift our emphasis away from a reliance on ‘metrics’ based solely around the academic citation of a research paper and its hosting journal (e.g. JIF), as supported by signatories of DORA ^30^.

Open peer review can play an important part in this, as researchers can gain visibility and credit for their contribution to the progression of another’s work. Furthermore, transparent refereeing provides researchers, and potential users of research, with another marker of quality as a peer reviewer’s credentials and what they say about a piece of research can become part of the assessment – instead of hidden and lost from the public record. A more appropriate use of citation-based indicators should also be included in measuring quality, such as the Relative Citation Ratio (RCR) recently proposed by the NIH ^31^, remembering that citation-based measures take considerable time, which may be an issue particularly for younger researchers. Post-publication identification of interest and importance of an article, and commentary about the context and potential implications of the findings should become a key aspect of science journals in the future, and could of course play a role in this qualitative assessment of research.

It is, of course, not usually possible to predict the longer term impact or consequence of a new discovery at the time of publication and refereeing (e.g. DNA fingerprinting, monoclonal antibodies), and so it remains important that reflections on the significance of research can be done at any time in the future (as is the practice on F1000Prime, PubMed Commons etc).

## What next?

Individual elements of what is described above have been developed by many groups. For example, there has been a steady rise in the use of open peer review since the launch of the medical BMC-series journals in the early 2000s followed by *BMJ Open*, both using mandatory open peer review, and others have followed suit more recently offering opt-out open review such as *Nature Communications*. However, because this process is still conducted before the article is made publicly available, the peer review history is only made visible for those articles that ultimately get accepted and still hides the reasoning behind any decisions to reject articles. The increasing discontent amongst researchers and the scientific community as a whole has given rise to new approaches such as *eLife*, and both they and PLOS have tried to make a stance against the JIF by vowing to never advertise their JIFs – this of course does not prevent the use of journal titles in making decisions that affect a scientists’ future. There has also been a rise in the prominence of data as a key element of publication, with the launch of data journals such as *GigaScience* and *Scientific Data*, and more stringent data policies for existing journals such as the *PLOS ONE*’s data policy, released in 2013 and adjusted in 2014.

The elements combined together into a single platform as described above has already been developed and is in active use by thousands of scientists through F1000. This combines the open science publishing platform F1000Research (the option to initially post an article as a preprint for general community comment will be added very shortly), with tools to assist in writing in F1000Workspace, and some measures of qualitative assessment of published articles through F1000Prime, both to inform reading and to help assess new findings after publication. Many other publishers have begun to create similar platforms that would compete to provide such services to researchers and funders.

However, a widespread change to a new way of publishing is unlikely to happen whilst the research community relies primarily on journals to provide the outlet for research findings. We therefore propose that to enable open science to succeed, researchers should be able to publish any research data and findings that they consider to be useful to others and to publish it without delay. To achieve this, we believe that there needs to be a fundamental shift in the way research findings are shared.

Publishers and others can support this process by providing services that meet a community-agreed set of rules (such as those suggested above); competition between providers will naturally lead to improved services and reduced costs. Meanwhile, journals could begin to provide qualitative assessment and to encourage discussion of findings published on these platforms, for example like *Nature* magazine’s News & Views section or *Current Biology*’s Dispatches. Funders are perfectly placed to help drive this shift by approving those providers that offer a high quality service that meet the agreed requirements. In time (as with open access), we anticipate that making research findings and data available in this open science way will ultimately become the norm and a requirement of all public funders. Researchers would then be free to choose whichever of the approved services they prefer, and articles would also be available to all on mirrored repositories (as open access articles are now available on PubMed Central and Europe PubMed Central), together with all their versions, referee reports and others comments, and the supporting data.

In the long term, it should be irrelevant where researchers publish their findings. What is important is that to speed up scientific progress, discovery and impact, research should be shared and made available without delay for others to use and to build upon. Making findings available needs to be done within a framework that encourages quality standards and requires all players in the research community to work as collaborators.
