# Supplementary material for: Towards an open science publishing platform
Source: F1000Res. 2016 Feb 3;5:130. [Version 1] doi: 10.12688/f1000research.7968.1 (PMC4768651; doi:10.12688/f1000research.7968.1)
Supplement: Peer review history

# Peer review: Referee response for version 1

**DOI:** 10.5256/f1000research.8575.r12629

**Published:** 2016-02-24

**Authors:** Gary G Borisy

**Affiliations:** 1 The Forsyth Institute, Cambrdge, MA, USA

The printing press, the disruptive technology of 400 years ago, revolutionized how science was communicated. Journals, along with editors and reviewers—the peer review process--eventually replaced an author-directed process of letter writing. A new disruptive technology has placed us in the midst of another revolution in communication. The power of the internet has enabled communication with a rapidity, connectivity and interactiveness unlike anything possible with print media. It therefore behooves the research community to rethink how the science communication process might be structured.

Tracz and Lawrence present to us their rethinking. They consider journals to be an outdated mechanism for publishing science and they enumerate the multiple problems familiar to researchers—the delay, the non-transparency, the tussles with reviewers and editors, the jockeying for high-impact journals. Tracz and Lawrence ask if journals are really necessary anymore. In so many other areas of 21 ^st^ century life, the internet has enabled the elimination of intermediaries between the end consumer and the producer. Are we at a similar stage for publications?

Tracz and Lawrence propose an Open Science Platform that enables authors to publish essentially immediately after they consider their work ready to be shared with the scientific community. The work is then subjected to a post-publication review process which is transparent because the reviewers are not anonymous and their critiques as well as the authors responses are posted as an integral part of the publication process.

A key issue is how the reviewers will be selected. Tracz and Lawrence propose that the authors be empowered to select and invite reviewers from a pool of qualified researchers. Of course, this shifts the question to how the reviewers become qualified and who makes this decision. The authors suggest that a virtual faculty such as that of F1000 could serve this function. Researchers would be incentivized to deliver thoughtful reviews not only because they would be identified but because their reviews would be citable documents in their own right. This would provide a benefit to reviewers which is lacking in the current system.  

A question that will spring to the lips of every researcher is how, in such a system, will we find the important publications. Tracz and Lawrence say that, in the long run, it should be irrelevant where authors publish their findings. But there are so many publications and so little time. Don’t journals with their editorial boards serve as key gatekeepers? Doesn’t the much-maligned impact factor nevertheless serve as a helpful guide? The Open Access movement has greatly facilitated the dissemination of research results but it has had the unintended consequence of also facilitating the emergence of predatory journals whose business model is to provide publication for pay with only the appearance, not the reality, of bona fide editorial quality control. This only compounds the problem.

Indicators of the importance of publications will remain necessary. The Open Science Platform does not, per se, provide the indicators but it does provide a framework in which quantitative and qualitative metrics of importance such as citations, downloads, blogs and commentary can be incorporated into the platform. In principle, these article-based metrics can be made discoverable and substitute for the classic journal gatekeepers.

Tracz and Lawrence have done the research community a service by proposing this bold, new Open Science Platform. Apprehensions aside, we have crossed into the 21 ^st^ century and there is no turning back.

I have read this submission. I believe that I have an appropriate level of expertise to confirm that it is of an acceptable scientific standard.

# Peer review: Referee response for version 1

**DOI:** 10.5256/f1000research.8575.r12434

**Published:** 2016-02-15

**Authors:** Johanna McEntyre

**Affiliations:** 1 Europe PubMed Central and EMBL-EBI, Cambridge, UK

This article outlines a new model to making scientific findings public. It argues that typical journal publishing processes are no longer fit for purpose and discusses pros and cons of preprints, before describing "Open Science Platform". The article is very timely given the latest discussions around preprints in biology, stimulated by Ron Vale's preprint, then article on the subject (Ref. 19 in this article).

Open Science Platform is in essence a nose-to-tail set of services that enable researchers to collaboratively write, publish, openly peer review, revise, credit, evaluate and archive scientific articles with minimal friction. The philosophy is that anything we think would be useful to publish should be published, and we will organize this bulk by devising better filtering/discovery mechanisms and reward systems post publication. While quite a few journals have touched many parts of this particular elephant, there is not to my knowledge an existing public platform that offers all these steps aside from the one described here. F1000 has developed such a platform, but the authors note that many other publishers are probably also building similar platforms to provide some healthy competition on the services provided.

I have no major criticisms of the article itself - it is not overly pushing a particular (F1000) product but rather painting a picture of what open science publishing might look like, reviewing challenges and possible solutions to the sub-optimal aspects of our current publishing systems. Frequent reference is made to many of the on-going efforts in this area and the authors clearly state their roles at F1000.

An important issue that was given only brief attention is the matter of data and other research objects associated with articles. Open science extends to many aspects other than article publishing. The authors acknowledged that data underling assertions in articles are critical, as is the need to deposit data in community-supported repositories with data-appropriate access, but delegates this particular challenge to other groups. Clearly research communities and resources need to lead from different angles - but nevertheless in a more rapid and expansive publishing mode, new mechanisms will be required to ensure rigour, and support the desired behaviour around data deposition and archiving. One of the key points of open science is to enable reuse of data and other outputs in other contexts. Therefore an open science platform for articles will need to operate in a well-coordinated fashion with data archives and processes to ensure that the gains in article publishing also apply to related data and other research objects (and the emerging credit systems for those too).

One aspect I would have liked to have seen the authors explore more is the fundamental matter of how to stimulate uptake of open science platforms by the key target audience: researchers. The technical challenges are tractable, the social ones the blocker. For many researchers, deeply engaged in their work at the bench, such open science systems are a long way from current operating practices, so adoption may take a while and many incentives along the way required.

I have read this submission. I believe that I have an appropriate level of expertise to confirm that it is of an acceptable scientific standard.
